# Spontaneous Cerebrospinal Fluid Rhinorrhea from a Prolactin-Secreting Pituitary Macroadenoma

**DOI:** 10.7759/cureus.13111

**Published:** 2021-02-03

**Authors:** Aneek Patel, Yair M Gozal, Hussam Abou-Al-Shaar, Philipp Taussky, William Couldwell

**Affiliations:** 1 Neurosurgery, New York University, New York, USA; 2 Neurosurgery, Mayfield Clinic, Cincinnati, USA; 3 Neurological Surgery, University of Pittsburgh Medical Center, Pittsburgh, USA; 4 Neurosurgery, University of Utah, Salt Lake City, USA

**Keywords:** prolactinoma, cerebrospinal fluid rhinorrhea, pituitary adenoma, cerebrospinal fluid leak, transsphenoidal surgery

## Abstract

Cerebrospinal fluid (CSF) rhinorrhea is a rare complication of macroprolactinomas that, in the vast majority of cases, is subsequent to either medical or surgical intervention. Here, we present the successful management of a rare case of spontaneous, noniatrogenic CSF rhinorrhea in a patient with an untreated macroprolactinoma. A 27-year-old man with no significant medical history presented with six months of persistent CSF rhinorrhea, which was confirmed by testing for beta-2-transferrin. He had had decreased libido since adolescence and impaired growth of secondary sexual characteristics. Workup revealed an elevated prolactin level, and imaging demonstrated erosion of the anterior sellar floor and soft tissue within the sphenoid sinus, concerning for tumor. The patient underwent surgical repair of the CSF leak via a transnasal transsphenoidal approach, with resection and biopsy of tumor material within the sinus. No tumor was noted within the sella itself. The patient tolerated the procedure well and had subsequent normalization of his prolactin level with no further CSF egress. Spontaneous noniatrogenic CSF rhinorrhea, although rare, should be considered in the differential diagnosis of invasive pituitary macroadenomas, especially prolactinomas. The mechanism of CSF leak from a prolactinoma is not completely understood, but the CSF leak should be urgently repaired through a transnasal transsphenoidal approach. Concurrently, tumor resection should be performed and a postoperative lumbar puncture or lumbar drain should be considered to reinforce the skull base reconstruction.

## Introduction

Prolactin-secreting adenomas originating from the pituitary gland account for 30-40% of all pituitary adenomas; however, macroprolactinomas (clinically significant prolactinomas >10 mm in diameter) make up a very small subset of these tumors [1-4]. Macroprolactinomas can lead to mass effect causing visual field deficits, cranial neuropathies, and hormonal dysfunction. Cerebrospinal fluid (CSF) rhinorrhea is a rare complication of pituitary macroadenomas that result either from CSF egress through a surgical defect or from erosion of the bony sella and dura by the tumor. Most commonly, the latter mechanism occurs after medical treatment of prolactinomas by dopamine agonists that leads to tumor shrinkage and exposure of the sellar defect. Here, we report the successful management of a rare case of CSF rhinorrhea as the presenting symptom for a prolactinoma with no prior medical treatment and review the relevant literature.

## Case presentation

A 27-year-old man with no significant medical history presented with a persistent severe headache and six months of constant rhinorrhea from his left nostril. He described the drainage as clear and tasteless. He also endorsed generalized fatigue, an inability to grow facial hair, and deficiencies with his libido since he was a teenager. He denied any prior occurrences of prolonged rhinorrhea, breast tenderness, or galactorrhea. His visual examination revealed fully intact visual fields and a benign funduscopic examination. The remainder of his neurologic examination was essentially normal. His prolactin level was elevated to 74.2 ng/mL, and the rest of his hormone panel was unremarkable.

**Figure 1:**
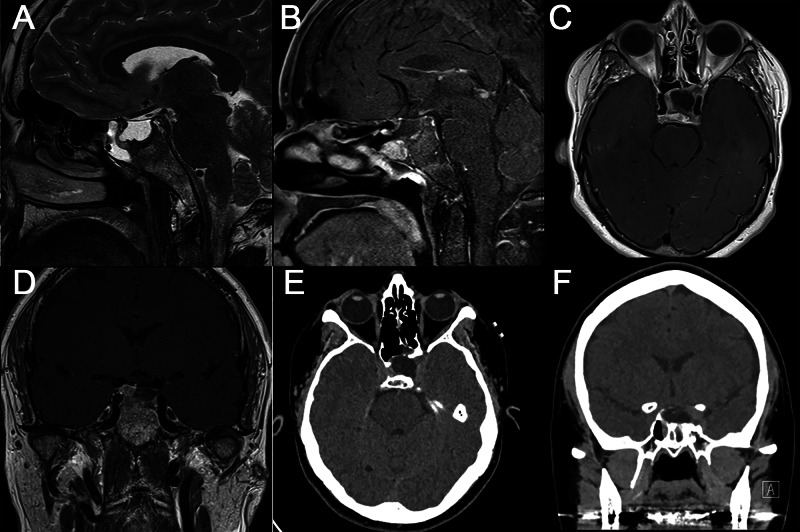
Preoperative sagittal T2-weighted (A), sagittal (B), axial (C), and coronal (D) contrasted T1-weighted MRI as well as axial (E) and coronal (F) CT scans showing CSF-filled sella, tumor in the sphenoid sinus, deviation of the pituitary stalk and gland, eroded anterior sella wall, and osseous destruction at the base of the sphenoid extending to the medial margin of the left cavernous sinus. MRI, magnetic resonance imaging; CT, computed tomography; CSF, cerebrospinal fluid

A sample of the nasal drainage tested positive for beta-2-transferrin, indicating an active CSF leak from the nostril. Magnetic resonance imaging (MRI) of the brain revealed an enlarged, CSF-filled sella and deviation of the pituitary stalk and gland towards the right. The anterior wall of the sella appeared eroded by a mixed cystic and solid 1.2 × 1.9 × 1.2-cm lesion within the sphenoid sinus along with CSF. Osseous destruction with sclerotic margins could be appreciated at the base of the sphenoid and extending to the medial margin of the left cavernous sinus (Figure 1).

The findings were concerning for a pituitary macroadenoma, likely a prolactinoma, that had been spontaneously evacuated into the sphenoid sinus. Given the risk of infection associated with persistent CSF rhinorrhea, surgical repair of the defect via a microscopic endonasal transsphenoidal approach with concomitant resection of any residual tumor was recommended. Upon opening the face of the sphenoid, the tumor was identified as a soft, off-white mass within the right side of the sphenoid sinus. The mass was resected by suction, and samples were sent for histopathologic analysis. The sphenoid septum was removed to increase exposure and ensure a complete resection. The sella was explored using the endoscope, but no evidence of tumor residual was noted. The anterior wall of the sella was found to be entirely dehiscent along with an absence of dura in the area. The arachnoid membrane had herniated into the sella, and clear CSF egress into the sphenoid sinus was observed. The sella was explored, but no evidence of tumor residual was noted. Closure of the sellar defect was then accomplished with the use of an autologous abdominal fat and fascia graft packed into the sphenoid sinus and bolstered with Surgicel strips (Ethicon, J&J Medical Devices, Somerville, NJ, USA) across the face of the sinus. A high-volume lumbar puncture was performed after closure to lessen the pressure on the repair. There were no intraoperative complications, and the patient was transferred to the critical care unit in stable condition. On postoperative day two, his prolactin level had decreased to 29.4 ng/mL. He was discharged in stable condition on postoperative day three and had no further recurrence of CSF rhinorrhea.

**Figure 2:**
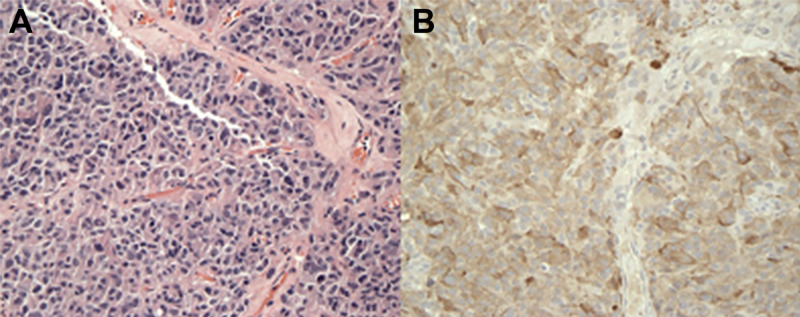
Immunohistochemistry of the pathological sample. (A) Hematoxylin and eosin staining of mass sample. Sheets of pleomorphic cells with rare mitosis can be appreciated. (B) Prolactin staining of the mass sample reveals diffuse staining.

Pathological examination of the resected sample revealed sheets of moderately pleomorphic cells with rare mitosis and no normal pituitary tissue. Immunohistochemistry revealed diffusely positive prolactin staining. Therefore, a diagnosis of prolactin-secreting pituitary macroadenoma was established (Figure 2).

## Discussion

Patients with prolactinomas commonly present with early hormonal manifestations of hyperprolactinemia and/or neurological manifestations imposed by tumor-imparted mass effect. Although it can result in galactorrhea, hyperprolactinemia in males manifests most appreciably as hypogonadotropic hypogonadism and decreased libido [5]. These signs were also evident in our patient, who reported having had low libido since his teenage years and deficiencies with secondary sexual development, including impaired body hair growth. However, it is not uncommon for symptoms to go unnoticed for long periods, leading to delayed presentation related to tumor mass effect and neurological sequelae in the form of vision changes, cranial nerve palsies, and headaches [3,6,7].

Spontaneous CSF leaks have been previously reported after medical management of prolactinomas, especially larger lesions [8,9]. Prolactin-secreting macroadenomas and giant prolactinomas are highly invasive, including into extrasellar spaces [2]. As they grow, they erode through bone and dura, intrinsically plugging any bony and meningeal voids they create in the process. Tumor shrinkage after medical management [10] can uncover areas of osseous and dural destruction, leading to unopposed CSF leakage [11,12], whereas spontaneous CSF leak without prior treatment, as was seen in our patient, is an extremely rare presentation for untreated prolactinomas with scarce reports in the literature. Moreover, in the current case, the majority of the patient’s tumor appeared to have spontaneously evacuated from the sellar region into the sphenoid sinus.

In the absence of medical or surgical intervention, the underlying pathophysiology of spontaneous CSF rhinorrhea is less clear. Ohtakara et al. [13] hypothesized three potential mechanisms for CSF leak in the setting of an untreated macroadenoma: a fistula between the subarachnoid space and the cavum sellae; a channel for CSF within the tumor itself; or a connection between the sella and a paranasal sinus. Other potential causes include rapid, spontaneous shrinkage of the prolactinoma secondary to intratumoral apoplexy or the formation of a CSF fistula secondary to an increase in intracranial pressure [14]. Imaging and intraoperative findings confirm invasion of our patient’s macroadenoma from the sella into the sphenoid sinus, providing a direct route into the nasal cavity for tumor evacuation and CSF rhinorrhea.

The presence of an empty-appearing sella on imaging in the setting of a persistent headache, rhinorrhea, and mild visual field defects also raised concern for idiopathic intracranial hypertension (IIH) and was therefore ruled out in this case. An empty sella is radiographically found in 77% of patients with IIH and represents elevated intracranial pressure compressing the pituitary gland along the sellar floor [15]. Atci et al. have demonstrated that 28% of IIH-caused empty sella can present with elevated prolactin levels, as seen in this patient [16]. However, IIH workup revealed no papilledema to suggest elevated intracranial pressure. Additionally, the presence of a mass on imaging provided a consistent explanation for the patient’s presentation.

In cases of spontaneous CSF fistula, rapid diagnosis and surgical intervention to repair the leak are critical regardless of the cause. Untreated CSF egress is associated with a high risk of meningitis, pneumocephalus, or development of an intracranial abscess [17]. The overall mortality rate for patients with untreated CSF rhinorrhea ranges from 25% to 50% [18]. Therefore, rapid and timely intervention, typically via a transnasal transsphenoidal approach, is essential. Preoperative planning for sellar reconstruction, either with the use of fat or fascial grafts or via nasoseptal flap reconstruction, is recommended.

## Conclusions

Spontaneous noniatrogenic CSF rhinorrhea, although rare, should be considered in the differential diagnosis of invasive pituitary macroadenomas, especially prolactinomas. The mechanism of CSF leak from a prolactinoma is not completely understood, but the CSF leak should be urgently repaired through a transnasal transsphenoidal approach. Concurrently, tumor resection should be performed, and a postoperative lumbar puncture or lumbar drain should be considered to reinforce the skull base reconstruction.
